# A Novel Canine Model of Acute Vertebral Artery Occlusion

**DOI:** 10.1371/journal.pone.0142251

**Published:** 2015-11-06

**Authors:** Yunfeng Zhang, Min Jin, Bin Du, Hao Lin, Chengyong Xu, Weijian Jiang, Jianping Jia

**Affiliations:** 1 Department of Neurology, Xuan Wu Hospital, Capital Medical University, Beijing, China; 2 The Second Artillery General Hospital of Chinese People’s Liberation Army, Beijing, China; 3 Department of Neurology, Affiliated Hospital of Nantong University, Nantong, China; University of Münster, GERMANY

## Abstract

**Background:**

The extended time window and theoretic reduction in hemorrhage make mechanical strategies an attractive approach for the treatment of patients with ischemic stroke. However, a limited availability of suitable animal models of cerebrovascular thrombosis has hampered the study of novel endovascular interventions. The aim of the present study was to develop a new technique for site-specific placement of a thrombus in a canine model that would allow for the evaluation of mechanical thrombectomy and clot retrieval methods and the visualization of thrombus dislocation or fragmentation during angiographic manipulation.

**Methods:**

Angiography and embolization with a preformed thrombus were performed in 12 canines. Under fluoroscopic guidance, an embolism protection device (EPD) was anchored to the middle segment of the left vertebral artery (VA) via the left femoral arterial sheath. A preformed radiopaque clot was injected through the guide catheter into the left VA, via the contralateral femoral artery, proximal to the EPD. After 15 min of occlusion, the EPD was removed and persistent occlusion of the VA was documented angiographically.

**Results:**

Angiography performed during the observation period confirmed the persistence of VA occlusion in each case, and displacement of the radiopaque clots did not occur during the 3-hour observation period. The technique allowed selective embolization of targeted vessels without thrombus fragmentation.

**Conclusion:**

This study demonstrates, for the first time, a canine model of post-circulation embolism induced by autologous blood clot placement. This model can be rapidly formed and easily operated, and the site of thrombosis can be readily controlled.

## Introduction

Stroke is one of the leading causes of morbidity and mortality worldwide, and its incidence has continued to increase each year [1]. The focus of the treatment of acute ischemic stroke (AIS) is to achieve rapid and effective revascularization to restore perfusion [2,3]. Presently, a well-accepted method for effective treatment of AIS is intravenous injection of recombinant tissue-type plasminogen activator (tPA) within 4.5 h of symptom onset [4]. However, intravenous administration of this thrombolytic agent is associated with a low recanalization rate and complications such as intracranial hemorrhage and systemic bleeding in patients with macroangiopathy and cardiogenic embolism, limitations that negatively affect the clinical efficacy of recombinant tPA [5].

The use of retrievable self-expanding stents for thrombectomy of acute proximal intracranial artery occlusions is a promising method for the treatment of AIS [6–11]. The extended time window and theoretic reduction in hemorrhage make mechanical strategies an attractive approach in patients with AIS, particularly in those in whom intravenous thrombolysis is ineffective or contraindicated [9,10]. Several recent clinical trials have demonstrated a clear therapeutic benefit of mechanical thrombectomy [12–15]. Nonetheless, further development and refinement of mechanical thrombectomy techniques would likely improve future patient outcomes. Various animal models of AIS have been developed using mice, rats, rabbits, sheep and pigs [16,17]. However, these preclinical models of cerebrovascular thrombosis are poorly suited to the study of mechanical thrombectomy and clot retrieval therapies due to limitations associated with the sizes and anatomic organization of the intracranial vessels, which differ substantially from those of humans [16,17]. The introduction of new animal models would not only facilitate advances in mechanical thrombectomy techniques, but also permit the evaluation of a wide range of other novel treatments for AIS, including both medical and surgical therapies.

In order to test emerging techniques, including endovascular therapy, in the treatment of AIS, it will be necessary to develop a clinically relevant model of site-specific thromboembolism. If the model is to be used in the assessment of endovascular therapy, it will need to be developed in a system large enough to accommodate endovascular devices, and must allow for targeted vessel occlusion to permit grading of the response to treatment. The present study was designed to assess the potential utility of a new canine model of segmental unilateral vertebral artery embolism that fulfills the aforementioned criteria. The data acquired indicate that this model yields highly reproducible results, and that its use may facilitate future development of new treatments for AIS, including novel mechanical thrombectomy and clot retrieval therapies.

## Materials and Methods

### Experimental animals

All procedures were conducted according to international guidelines for animal experimentation, and the protocol for the study was approved by the Animal Ethics Committee of Beijing Pinggu Hospital, Capital Medical University, Beijing, China. All effort was made to alleviate suffering during the surgical procedures and post-surgery recovery, and none of the animals were sacrificed during the experimental period. Six male and six female adult mongrel dogs, with a mean body weight of 20.9 ± 1.5 kg (range 20–25 kg), were used in this study. Free access to food and water was given until the night before angiography; the animals were fasted overnight (for both solids and liquids) before surgery. One day before percutaneous intervention, the dog received a 300-mg loading dose of clopidogrel. On the day of surgery, the animal was sedated with telazol (6 mg/kg body weight; volume < 3 mL; total dose < 150 mg; Zoetis Inc., Kalamazoo, MI, USA) administered by intramuscular injection. Endotracheal intubation was performed and the animal mechanically ventilated (12 breaths/min; volume 5 mL/kg). General anesthesia was maintained by continuous inhalation of 1.5–2.0% isoflurane (Baxter Deerfield, IL, USA). Continuous monitoring of vital signs, including heart rate (via the electrocardiogram), blood pressure, expired carbon dioxide level and body temperature, was performed, and these variables were maintained within the physiologic range.

### Thrombus preparation

A 20-mL syringe was connected to the arterial sheath in order to collect a 20-mL sample of whole blood. After the addition of 2 g of barium sulfate (Sigma, St. Louis, MO, USA), the syringe was gently swirled for 10–30 s, allowing barium sulfate to deposit naturally and preventing barium-induced anticoagulation. The syringe containing the whole blood sample was placed vertically at room temperature for 2 h, until the different blood components had stratified. The serum was discarded and the remaining solid was split into two layers, with the upper layer enriched with fibrin. The syringe was unplugged and the contained blood clots were poured out. The blood clots were surface-dried with gauze, and the upper-layer thrombi were cut carefully into small pieces (approximately 3 mm × 20 mm) using a No. 11 surgical blade. The small pieces of thrombi were squeezed into the end of a 6F guiding catheter filled with normal saline, which was then connected to a tee and syringe for use.

### Model establishment

The animal experiments were conducted in the experimental animal base of Beijing Pinggu Hospital, Capital University of Medical Sciences. The canine model of segmental unilateral vertebral artery embolism was established with the aid of WINMEDIC-2000 digital subtraction angiography apparatus (Lepu Medical Technology, Beijing, China). After puncture of the bilateral femoral arteries, 6F and 8F arterial sheaths (Terumo Corp., Tokyo, Japan) were placed, with continuous intrathecal flushing using heparin saline (10 U/mL; Jiangshan Pharmacy, Jiangsu, China). Under X-ray guidance, a 6F guiding catheter was introduced through the 6F arterial sheath into the initial segment of the left vertebral artery. Angiography was performed using manual injection of Omnipaque contrast agent (iohexol; injection volume 6 mL; GE Healthcare Inc., Princeton, NJ, USA) through the 6F guiding catheter.

A custom-designed, dedicated, self-expanding thrombus filter (length 10 mm, diameter 4 mm; Hunan Ruikangtong Technology Development Co., Ltd., Changsha, China) was pre-installed in the outer catheter of a delivery/retrieval catheter. The thrombus filter was introduced through the guiding catheter into the middle segment of the left vertebral artery, and then released to adhere to the vertebral artery wall. The filter wire was fixed, and the 6F guiding catheter was withdrawn into the aortic arch.

An 8F guiding catheter was introduced through the contralateral 8F arterial sheath into the left vertebral artery, with the head end positioned approximately 2 cm from the proximal end of the thrombus filter. The 6F guiding catheter, pre-installed with autologous thrombi, was introduced into the 8F guiding catheter. The thrombi contained in the syringe were slowly injected into the vertebral artery until they were proximal to the thrombus filter. Occlusion of forward blood flow was confirmed by angiography. After allowing the exogenous emboli to fully implant for 15 min, the 6F guiding catheter was withdrawn from the 8F guiding catheter. Angiography confirmed that the vertebral artery remained occluded. Then, the thrombus filter distal to the emboli was taken into the delivery catheter and slowly withdrawn from the body. After 45 min, angiography was performed to examine whether the canine model of acute vertebral arterial occlusion had been established successfully; the criterion for success was that the left vertebral artery remained occluded.

### Radiographic assessment

A blinded assessment was performed by two specialists in medical imaging that were non-members of the experimental group. Blood flow was evaluated using Thrombolysis In Myocardial Infarction (TIMI) flow grades: grade 0, no forward blood flow in the occluded segment; grade 1, contrast agent filling of the occluded segment with no development of a distal vascular perfusion area; grade 2, slow complete filling or rapid partial development of the distal vascular perfusion area; and grade 3, rapid complete filling of the distal vascular perfusion area [18].

### Statistical analysis

All data are presented as the mean ± standard deviation, and plotted using Office Excel software (Microsoft Corp., Redmond, WA, USA). Pair-wise or inter-group comparisons were performed using Student’s *t*-test. Differences were considered statistically significant at *P* < 0.05.

## Results

### Establishment of vertebral artery occlusion

**Fig 1:**
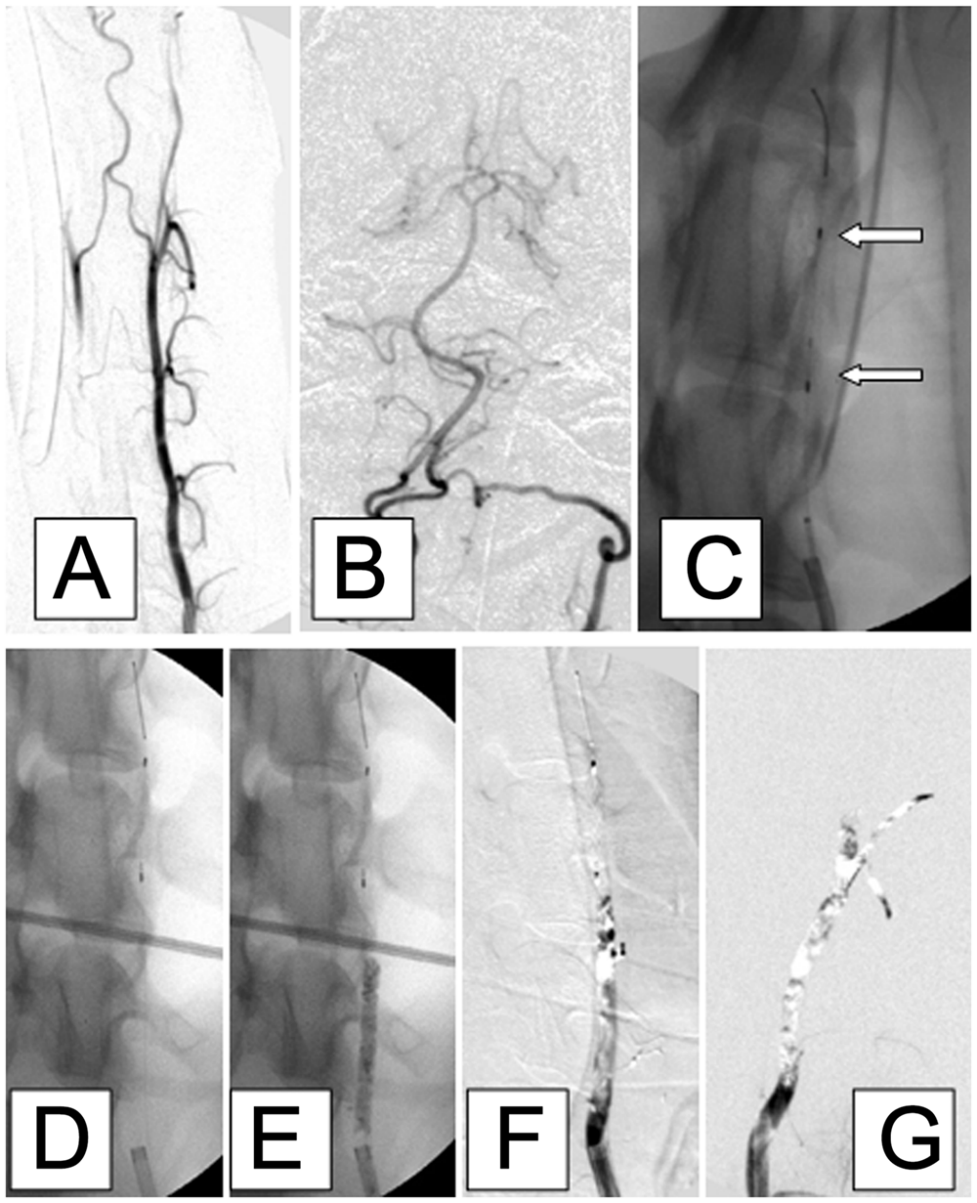
Representative angiographic images from a dog showing the successful establishment of left vertebral artery occlusion. A, B: Antero-posterior images of the left vertebral artery after injection of contrast. C, D: A custom-designed, dedicated, self-expanding thrombus filter (length 10 mm, diameter 4 mm; white arrows, panel C) was pre-deployed into the middle segment of the left vertebral artery. E: The non-subtracted image shows adequate radiopacity of the injected barium sulfate-marked thrombus. F: Digital subtraction image showing complete occlusion of the left vertebral artery. G: Complete occlusion of the vessel was confirmed 45 min later. Vertebrobasilar embolism did not occur distal to the thrombus filter.

The model of acute vertebral artery occlusion was established successfully in all 12 dogs, with the thrombi anchored at the target position, *i*.*e*., proximal to the thrombus filter. The mean length of the occluded segment of the left vertebral artery was 1.55 ± 0.05 cm, and did not differ significantly between female (1.56 ± 0.05 cm) and male (1.54 ± 0.05 cm) dogs (*P* = 0.39). Fig 1 presents a representative series of angiographic images from one animal demonstrating successful establishment of left vertebral artery occlusion. After injection of contrast (Fig 1A and 1B), the custom-designed self-expanding thrombus filter (length 10 mm, diameter 4 mm) was pre-deployed into the middle segment of the left vertebral artery (Fig 1C and 1D). Radiopacity of the injected barium sulfate-marked thrombus is shown in Fig 1E. Complete occlusion of the left vertebral artery was confirmed with digital subtraction imaging (Fig 1F), and the occlusion was still present 45 min later (Fig 1G). Vertebrobasilar embolism did not occur distal to the thrombus filter.

### Complications

No animals exhibited evidence of any complications, such as arterial vasospasm, dissection or perforation, related to the surgical procedure or use of equipment. Perioperative mortality was zero, and all dogs survived the course of the study.

## Discussion

The present study has described a novel technique for the site-specific placement of a thrombus in the vertebral artery of the dog. This canine model could potentially be used to assess novel medical and/or surgical therapies for AIS, and is particularly well suited to the evaluation of mechanical thrombectomy and clot retrieval methods, since it permits the visualization of thrombus dislocation or fragmentation during angiographic manipulation. This model can be rapidly and easily established, and the site of thrombosis can be readily controlled. Hence, this preclinical model of AIS could prove highly useful, facilitating the development and testing of novel treatments for AIS, including new methods for mechanical recanalization of arteries.

A variety of animals have been used as preclinical models of AIS, including mice [19], rats [20–22], rabbits [23,24] and sheep [25,26]. However, these animal models are poorly suited to the study of mechanical thrombectomy and clot retrieval techniques due to the small sizes of their arteries, or to an anatomic organization of their intracranial vessels that differs from that found in humans [16,17]. Previous *in vivo* studies of peripheral vascular interventional devices have largely used animal models established in miniature pigs [27–31]. However, the intracranial blood supply of miniature pigs is achieved mainly through the re-converging of a plexiform vascular network into the intracranial segment of the internal carotid artery. The vertebrobasilar arterial system is poorly developed in pigs, and as a result it is difficult to propel endovascular devices directly into an intracranial artery through the vascular network. For this reason, animal experiments that have focused on the efficacy of mechanical thrombectomy devices for acute intracranial arterial occlusions have primarily used the miniature pig model of external carotid artery branch occlusion developed by Gralla *et al*. [28]. This miniature pig model can only partially simulate embolism in terms of medical imaging, and it differs in important aspects from cerebral stroke in humans caused by occlusion of an intracranial artery.

The present study has established a new canine model of segmental unilateral vertebral artery embolism, which better simulates a posterior circulation ischemic stroke than a miniature pig model. Adult mongrel dogs with an approximate weight of 20 kg were used as the experimental animals, as their body size was considered suitable for vascular interventional studies. The femoral artery of the dog has a moderate diameter and a superficial position, facilitating direct puncture and sheath insertion by Seldinger’s technique. The left vertebral artery branches off directly from the left subclavian artery, and has a short course with few branches; thus, it is technically simple to approach the target segment of the vessel. The canine circle of Willis is well developed, and supplies the anterior and middle cerebral arteries of the internal carotid arterial system through the posterior communicating artery. Compared with the vascular anatomy of the miniature pig, the vascular arrangement in the dog bears a much closer similarity to that found in humans. Additionally, the well-developed canine vertebral arterial system rarely undergoes vasospasm, dissection or perforation, and its diameter is approximately 3 mm, similar to the diameters of arteries in the human vertebrobasilar system. The canine vertebral artery has the anterior spinal artery as a direct branch, and then continues directly to the basilar artery and posterior cerebral artery. Thus, it is feasible to observe embolus movement and the occurrence of distal embolism through fluoroscopy.

Owing to the above advantages, adult dogs can serve as an ideal experimental animal model for simulating a posterior circulation infarct. However, the canine vertebral artery is well developed and has a strong blood flow, which makes it difficult for exogenous emboli to segmentally anchor to the artery on one side. It is for this reason that few previous studies have reported a canine model of vertebral artery occlusion. The present study successfully developed a distal thrombus filter that enabled exogenous emboli to fully attach to the vessel wall without being dislodged due to the impact of the blood flow. The novel device was also able to control the length of the external embolism that was injected into the target vessel. After tight occlusion of the target vessel through secondary thrombosis, the filter distal to the thrombi is withdrawn through a micro-catheter, while the exogenous emboli remain in the target vessel segment. In this way, an ideal animal model of a unilateral vertebral artery embolism is established. Although canine models have been described previously [32], to the best of our knowledge this is the first report in the literature of a technique that uses a distal thrombus filter to control the siting and length of the thrombus.

The canine model of vertebral artery occlusion described in this study could potentially be used to evaluate a variety of novel therapies for AIS, both medical and surgical. In future studies, the assessment of treatment efficacy could be achieved through the use of various outcome measures, including neurobehavioral tests (such as the canine stroke scale) [33], imaging studies (such as magnetic resonance imaging and 2,3,5-triphenyltetrazolium chloride staining to establish infarct volume and lesion location) and mortality rates. If required, sham surgery could be readily performed by omitting the insertion of the thrombus filter and injection of thrombi into the vertebral artery.

A limitation of this research is that vascular pathology was not investigated in order to clarify the safety of the model establishment regarding vascular endothelial damage. However, since angiography data demonstrated no occurrence of thrombosis, arterial dissection or arterial perforation after recanalization, it would appear that the device is clinically safe and minimally invasive.

Segmental unilateral vertebral artery embolism in the canine represents a highly reproducible model for site-specific vascular thromboembolism that is well suited for the introduction of interventional devices. This model will prove highly useful in the evaluation of novel medical and surgical therapies for AIS, and is particularly suited to the testing of new devices designed for interventional therapy of stroke.

## Supporting Information

S1 Data Raw data for the 12 dogs included in the study. The age, gender, weight and color of each animal are provided, together with details of the embolic position, blocked blood vessel diameter, embolus length, and blood flow classification before and after thrombogenesis.
(XLS)
